# Navigating the Genetic Landscape: Investigating the Opportunities and Risks of Cross-Species SNP Array Application in Catfish

**DOI:** 10.3390/genes16060717

**Published:** 2025-06-18

**Authors:** Bettina Hegedűs, Zoltán Bagi, Szilvia Kusza

**Affiliations:** 1 Centre for Agricultural Genomics and Biotechnology, Faculty of Agricultural and Food Sciences and Environmental Management, University of Debrecen, Egyetem tér 1., 4032 Debrecen, Hungary; hegedus.bettina@agr.unideb.hu (B.H.); bagiz@agr.unideb.hu (Z.B.); 2 Doctoral School of Animal Science, University of Debrecen, Böszörményi út 138., 4032 Debrecen, Hungary

**Keywords:** cross-species genotyping, genetic diversity, marker-assisted selection, *Siluriformes*, SNP array validation

## Abstract

Aquaculture has become a crucial component of global food production, yet catfish (10.8% of global finfish production) breeding programs often lack sufficient genetic data to fully utilize their production potential. In the last 15 years, there have been improvements in this field as two high-density (HD) single nucleotide polymorphism (SNP) arrays (250K and 690K) and low-density panels have been developed for North American channel catfish (*Ictalurus punctatus*) and blue catfish (*Ictalurus furcatus*). This lack of genomic tools hinders genetic improvement efforts in other commercially relevant catfish species besides them. Therefore, this review investigated the reason behind the lack of SNP chip usage in genetic-based selections in most catfish breeding programs and the cross-species applicability of the already existing high-density SNP arrays for genotyping members of the *Clariidae*, African catfish (*Clarias gariepinu*), and *Siluridae*, European catfish (*Silurus glanis*), families. This paper systematically reviews the literature of more than 16 SNP arrays, with 66 non-target species, and assesses the possibility of adapting catfish SNP arrays to the catfish families of interest. With lowered filtering (e.g., MAF > 0) thresholds, the Affymetrix Axiom 250K and Axiom Catfish 690K Genotyping Array could potentially be used on important market species like African and European catfishes. In the long term, chip development would be the solution for these species, but, until then, cross-application is a viable alternative. Despite low polymorphic SNPs (~1%) and call rates (~0%), this SNP array could aid researchers and breeders, improving catfish aquaculture and management.

## 1. Introduction

*Siluriformes*, encompassing 40 families over 3730 described species and an estimated 1750 yet-to-be-described taxa, constitute a highly diverse clade within the ray-finned fishes. Catfishes exhibit global distribution, primarily inhabiting freshwater environments, although they are also present in coastal regions of continents and adjacent islands. Their distribution is mainly concentrated in the tropical regions of South America, Africa, and Asia. Catfish represent a significant component of global fish diversity, comprising approximately 12% of all teleost fishes [1,2,3].

Catfish aquaculture plays a crucial role in global food security, representing 3.57% of all produced (185.4 Mt) aquatic animals in 2022, which means they are the second most produced species group [4]. Channel catfish (*Ictalurus punctatus*), blue catfish (*Ictalurus furcatus*), African catfish (*Clarias gariepinu*), and European catfish (*Silurus glanis*) species have every key characteristic for aquaculture. They have a fast growth rate (rapid growth to market size), adaptability to captivity (excellent stress tolerance and resistance), efficient feed conversion (reducing feed costs), omnivorous diet (simplifies feeding management), and good meat quality (market demand) [5,6].

In the market, hybrids are often used as final products, but, despite the high hybridization potential of *Siluriformes*, documented hybrid crosses within them are limited, encompassing only 80 crosses in 11 families. Even while -omics approaches have been applied to catfish hybrids in aquaculture [7], the possible negative impacts of hybridization, especially in the case of invasive species, have not been addressed adequately. This lack of data hinders a complete understanding of the role of hybridization in catfish evolution and conservation. This underscores the need for further genomic research to clarify the evolutionary history of catfish and assess the ecological and economic consequences of anthropogenic hybridization [3,5,8].

Selective breeding programs should enhance production efficiency and desirable traits. However, the application of advanced genetic improvement strategies in these species trails considerably behind that observed in other prominent aquaculture model species, such as Atlantic salmon (*Salmo salar*), rainbow trout (*Oncorhynchus mykiss*), and Nile tilapia (*Oreochromis niloticus*) [9,10,11,12]. These programs aim to improve commercially important characteristics while relying on understanding genetic diversity and implementing appropriate selection methods [10]. Therefore, selective breeding programs are essential for enhancing production efficiency and desirable traits, such as improving commercially important characteristics like growth rate, disease resistance, and meat quality. Successful breeding strategies rely on a clear awareness of genetic diversity and the effective application of molecular genetics and selection methods [13,14,15,16]. SNP (single nucleotide polymorphism) chips are instrumental in advancing both aquaculture breeding programs and species conservation efforts. In breeding, they facilitate genomic selection (GS) and marker-assisted selection (MAS), enabling breeders to identify and select individuals with superior genetic traits [17,18]. From a conservation perspective, SNP chips provide valuable insights into genetic diversity, population structure, and evolutionary history, helping to identify endangered populations, develop effective conservation strategies, and monitor genetic diversity within captive populations. Species-specific SNP tables for aquaculture species are limited. This review explores the reason behind the lack of SNP genotyping in catfish species and the potential cross-species utility of an existing 690K SNP array designed for the channel catfish and blue catfish for genotyping African catfish and European catfish, species of significant aquaculture importance.

## 2. Literature Review Methods

The literature reviews used for this research were conducted in March 2025 and focused on two primary themes: conducting a SWOT analysis for SNP-chip-based genotyping and exploring the application of SNP chips to non-target species. For both themes, we examined four major databases: Google Scholar, PubMed, Semantic Scholar, and Web of Science (WOS), which are web search engines that specifically search scientific literature and scholarly resources. To filter the literature, we used the Publish or Perish software v.8 [19] for the first three databases, while for WOS, we utilized its native search engine. In both cases, the search results were initially narrowed down to articles published or available for early access from 2010 to 2025. Subsequently, the first 100 articles from each database were manually reviewed for further analysis. We reviewed various academic materials, including scientific articles, books, book chapters, conference proceedings, abstracts, dissertations, and scientific/official reports. However, multiple instances occurred where individual sources were repeatedly listed in the results, and other instances occurred where the results were not relevant to the objectives of our study. These irrelevant materials were excluded from further consideration.

A SWOT analysis was conducted using keywords related to “SNP chip,” “SNP array,” “genetic,” “breeding,” and “fish” focusing on “strength”, “weakness”, “opportunity”, and “threat”. Out of 102,517 sources, 1011 were reviewed, resulting in 549 qualifying publications. However, these were not exclusively related to animal species. Specifically, approximately 30% of the publications also included references to plants and/or humans. Among the filtered results, a total of 384 were identified that focused solely on animals. After additional filtering, 316 publications on fish species were identified, covering 75 unique species across 57 genera and 40 families, with 27 deemed most relevant for the review.

**Figure 1:**
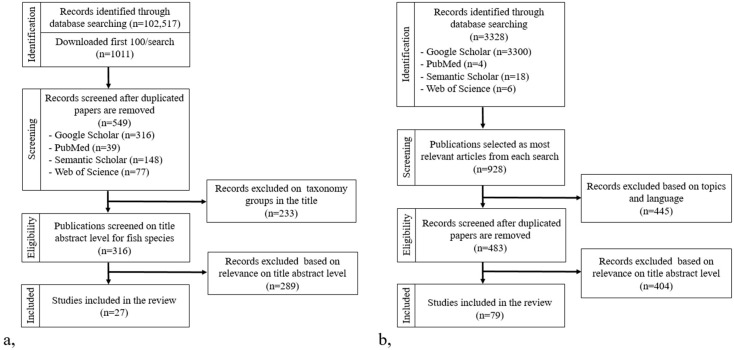
This PRISMA flowchart ((**a**), SWOT analysis; (**b**), SNP array) illustrates the steps conducted in this systematic review.

The study examined SNP array applicability by searching for specific keywords related to “SNP chip,” “SNP array,” and various taxa (bovine, catfish, canine, caprine, chicken, equine, ovine, salmon, and trout). After applying Publish or Perish software, 483 unique articles were selected. A manual screening process resulted in a final review of 79 relevant pieces of literature, including articles and reports, with additional references examined for deeper insights (Figure 1).

## 3. SNP Chips and Their Importance in Genetic Research

With the genetic and biotechnological revolution of the last decades, several technologies arose such as next-generation sequencing (NGS) (e.g., restriction site-associated DNA sequencing—RAD-Seq, Illumina short-read sequencing (Illumina, Inc., San Diego, CA, USA), and PacBio long-read sequencing (Pacific Biosciences, Inc., Menlo Park, CA, USA) and third-generation sequencing (Oxford Nanopore) (Oxford Nanopore Technologies plc, Oxford, UK), where whole genomes can be sequenced in a week, cost-effectively [20,21]. While whole-genome sequencing offers comprehensive genetic information and becomes more affordable for studies, lower-coverage sequencing and SNP arrays (SNP microarrays) are often combined with imputation and are frequently used for cost-effectiveness in large-scale genetic studies. SNP microarray (SNP chip) technologies find applications in many other areas [17]. Their main advantage is that they can safely and parallelly identify several hundred thousand different SNPs on a small surface. With this, they can genotype with a customized SNP array a selected taxonomic group (e.g., family and species) with different densities for several possible aims (e.g., for forensics, functional studies, quantitative trait loci (QTL) mapping, genome-wide association studies (GWASs), genomic selection (GS), and inbreeding studies) time- and cost-effectively [9,17,22,23].

Unfortunately, these arrays are not available for all species. However, they are primarily made for model organisms that are studied for a broad approach (e.g., *Gallus gallus domesticus*) or with species that are of production (e.g., *Bos taurus*) or ecological importance (e.g., *Rangifer tarandus*) [23,24,25]. Using an existing SNP panel for a non-model species can be more efficient than developing a new one [26]. One of the primary reasons for this is that designing a new SNP chip is costly and time-consuming, especially for non-model species, which often lack sufficient genetic information, such as a reference genome. Meanwhile, if an already existing SNP array is used, it can be applied immediately with known and validated performance characteristics and can provide information for comparative studies. Because of these reasons, there have been several occasions when cross-species applications were needed with SNP chips [27]. The arrays have inherent limitations in these cases because they were not specifically designed for that particular species or purpose. As a result, coverage, genotype call rates—which decrease by 1.5% for every million years of evolutionary divergence—and minor allele frequency (MAF) can be low [28].

## 4. Aquaculture of Catfish: SNP Genotyping in Breeding Programs

### 4.1. Implementation of Genetic-Based Selection of Catfish

Catfish aquaculture uses various breeding technologies, from traditional methods like mass and family selection to advanced molecular techniques. While traditional approaches rely on phenotypic observations, the channel catfish (*I. punctatus*) stands out due to its integration of SNP genotyping-based breeding programs, supported by substantial investment and research [10]. Decades of research, particularly in the United States, have led to the development of high-density SNP arrays specifically for channel catfish [29,30]. This species benefits from large-scale production and market demand, driving sophisticated genetic selection strategies [7]. Although genetic improvement is prominent in channel catfish, it is also relevant for other commercially important species, including the African catfish (*C. gariepinus*) and various Asian catfish [14,15]. Overall, the push for enhanced production efficiency and sustainability leads to broader adoption of genetic selection in catfish aquaculture.

The limited adoption of SNP genotyping in catfish aquaculture, excluding channel catfish, arises from a confluence of economic, technical, and biological factors. The absence of well-developed, species-specific SNP arrays restricts the applicability of this technology, hindering its effectiveness across diverse catfish populations.

### 4.2. SWOT Analysis of Genetic-Based Selection Frequency in Catfish Aquaculture

The adoption of genetic selection methods varies widely among species and production scales, but the utilization of SNP arrays in breeding programs is not widespread in the order *Siluriformes* [29,30]. A SWOT (Strengths, Weaknesses, Opportunities, and Threats) analysis provides a useful framework for assessing the factors that influence this trend, as observed in other aquaculture areas [31].

Modern genetic selection methodologies exhibit significant efficacy in the field of agriculture, particularly through the application of SNP array technology, which is accessible for the *I. punctatus*, in the case of catfishes [29,30]. This technology has the potential to drive significant genetic advancements in critical aquaculture traits, thereby enhancing both productivity and profitability [17]. Furthermore, ongoing investigations into WGS are instrumental in identifying novel genetic markers, which serve to bolster SNP-based selection strategies [32].

New genetic selection approaches in aquaculture encounters several constraints. A primary challenge now is the significant cost associated with SNP genotyping (e.g., infrastructure development), which may be prohibitive for small-scale enterprises [33]. Furthermore, the absence of comprehensive genomic resources for non-model species, such as the European catfish [34], hinders the establishment of targeted genetic improvement strategies [17]. Many aquaculture facilities also lack the bioinformatics and statistical genetics knowledge that is essential for the effective utilization of genomic data [35]. Additionally, an excessive emphasis on a limited number of elite individuals within breeding programs may result in a reduction in genetic diversity, thereby jeopardizing the long-term adaptability and resilience of the population [22].

In addition to the previously mentioned high costs, their decline related to technological advancements is making these methodologies gradually more accessible to aquaculture producers [35]. Furthermore, the development of genomic resources for non-model catfish species will enable customized SNP arrays, enhancing genetic selection applications [36]. The integration of genetic selection with advanced breeding technologies holds the promise of accelerating genetic improvements in aquaculture. Additionally, the rising consumer demand for sustainably produced goods is motivating producers to implement innovative genetic selection techniques aimed at enhancing efficiency and minimizing environmental impact [37].

Despite the existence of potential opportunities for advancement, several threats must be comprehensively addressed. The emergence of new pathogens and parasites has the potential to undermine existing resistance markers, necessitating continuous adaptations in breeding programs [38]. Moreover, environmental changes, including shifts in climate, can significantly impact the traits of genetically selected catfish, highlighting the necessity for flexible management strategies [39]. Additionally, regulatory complexities surrounding genetic technologies in aquaculture may impede implementation efforts, while negative public perceptions regarding genetically modified organisms can create market-related obstacles. Furthermore, prevailing misunderstandings among consumers regarding the distinctions between genetically modified organisms and those produced through selective breeding further complicate market acceptance [40,41].

To address one of the weaknesses (Table 1), this paper will further review the possible application of the catfish SNP array to economically important non-target species.

## 5. Similarities and Differences Among the Four Studied Catfish Species

Catfishes from the *Siluriformes* order are a diverse group with significant economic and ecological roles [42,43,44]. While they share traits like bottom-dwelling habits, carnivorous diets, nocturnal activity, scale-less bodies, and barbels, they also exhibit unique evolutionary adaptations and genetic differences [5] (Table S1). Based on these, while catfish can be suitable for aquaculture, they present risks as an invasive species [45,46,47].

The channel catfish (*I. punctatus*), a member of the *Ictaluridae* family (North American catfishes, diverged ~ 65–71 Ma), is a cornerstone of aquaculture, particularly in the United States [48,49]. Its hybrid with the blue catfish, known as the CB hybrid, offers advantages like superior growth and disease resistance [7]. Domesticated in the United States over a century ago [50], its global cultivation has increased [44]. Selective breeding, including efforts at USDA-ARS WARU, further enhances its aquaculture potential [10].

The blue catfish (*I. furcatus*), related to the channel catfish, shares similar habitats and behaviors [51,52]. While juveniles are hard to distinguish, mature individuals vary in size and morphology (Table S1). Valued for food and recreation, the rapid growth and invasive potential of blue catfish require control measures in some areas [52].

The African catfish (*C. gariepinus*), belonging to the *Clariidae* family (airbreathing catfishes), is another globally significant aquaculture species. The species originated in Southeast Asia and reached Africa about 40 Ma [48,53]. It has common catfish traits but features a unique gill structure for air-breathing, adapting to swampy environments [54]. Its commercial culture has grown significantly since 1970, especially in Asia [46]. However, genetic information and marker-assisted selection for this species and its hybrids, like “Claresse,” are still limited [46,55].

**Figure 2:**
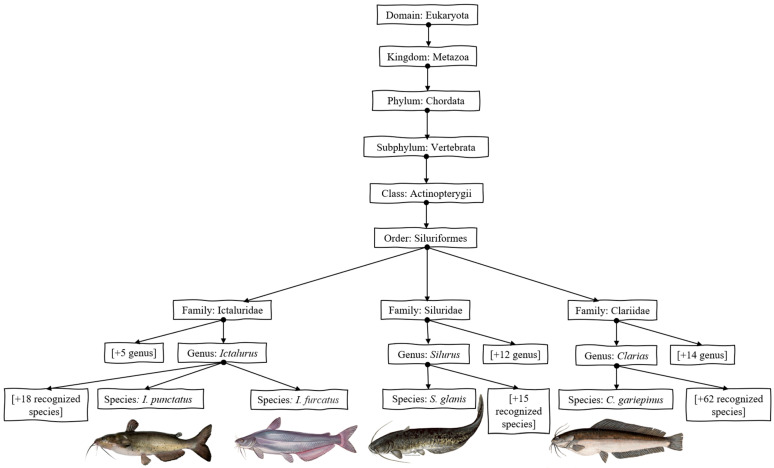
Phylogenetic relationship of channel catfish (*Ictalurus punctatus*), blue catfish (*Ictalurus furcatus*), African catfish (*Clarias gariepinu*), and European catfish (*Silurus glanis*) (database: [61]).

The European catfish (*S. glanis*), a member of the *Siluridae* family (diverged from *Cranoglanididae* ~ 49–66 Ma), is a significant aquaculture species in Europe, cultivated for over a century. Its adaptability and large size enhance its commercial and recreational value [56,57]. Improved aquaculture practices have boosted production efficiency and sustainability [58]. Since the 1800s, the European catfish has become important for food production and sport fishing [59,60]. These diverse catfish species, each with unique characteristics and evolutionary histories (Figure 2), contribute significantly to global aquaculture and fisheries.

## 6. SNP Arrays in Catfish and Its Application for Genetic Studies

Despite the large size of the *Siluriformes* (catfishes) order, most of the genetic information about the species is limited, and their genetic potential is not being exploit in the aquaculture. SNP arrays have proven to be effective tools for genome-scale genotyping in various fish species, including *S. salar*, *Cyprinus carpio*, *Paralichthys olivaceus*, and *O. mykiss* [9,62,63,64].

### 6.1. The First High-Density 250K Catfish Array

For catfish research, the initial tool was the Affymetrix Axiom 250K SNP array (Affymetrix, Inc., Santa Clara, CA, USA), developed without a reference genome, which necessitated an in silico analysis by Affymetrix. They evaluated 641,489 SNPs using a random forest model to determine probe performance, retaining only those with a p-convert value over 50%. Ultimately, 250,113 SNPs were included after excluding A/T and C/G SNPs. Validation involved genotyping wild channel catfish and various species, including blue catfish, brown bullhead (*Ameiurus nebulosus*), and white catfish (*Ameiurus catus*) [29] (Table 2). While the array demonstrated high conversion rates across species, the polymorphism rates were lower, indicating potential inbreeding in the D&B blue catfish strain. The study suggests that polymorphism rates may be underestimated due to a small sample size.

The 250K SNP panel facilitated the creation of a high-density genetic linkage map in a hybrid catfish system, consisting of 26,238 SNPs across 29 linkage groups and 12,776 marker positions [65]. This SNP array was crucial for GWAS studies on various traits. One study focused on heat stress tolerance in catfish, using interspecific backcross offspring from channel catfish females and F1 CB hybrid males. A GWAS on 556 progenies revealed a significant genomic region of about 1.0 Mb in linkage group 5 associated with body weight, benefiting breeding programs [66,67]. Another GWAS on F1-hybrids identified QTLs related to head size in catfish fingerlings, with genes linked to bone development found in these regions [68]. The 250K SNP catfish chip was utilized with a newly developed method for GWAS analyses (GRAMMAR-Lambda) to explore the genetic basis of head size traits in catfish [69].

### 6.2. The Axiom Catfish 690K Genotyping Array

The Axiom Catfish Genotyping Array (Affymetrix, Inc., Santa Clara, CA, USA) represents a significant advancement in SNP array technology, achieving 98.6% coverage of the new catfish reference genome [30,70]. A custom bioinformatics pipeline was utilized to curate SNPs based on specific selection criteria, including bi-allelic SNPs, a MAF greater than 10%, and GC content between 30% and 70%. Ultimately, 690,662 SNPs, consisting of 238,484 genic and 452,178 intergenic variants, underwent in silico probe conversion testing, resulting in a total of 693,567 probes. The array, similar to the 250K chip, features SNPs from channel catfish, blue catfish, and interspecific populations (Table 3), along with 48,434 SNPs for strain identification in channel catfish aquaculture and 6622 SNPs from a wild Coosa River population. Additionally, 2000 quality control probes were included. The genic SNPs originated from 24,186 annotated channel catfish genes or assembled RNA-Seq transcripts. The array exhibited high quality, with an average p-convert value of 0.71 and 89.6% of SNPs showing a MAF exceeding 10%, confirming its suitability for various genetic analyses.

Approximately 95.5% of the SNPs on the array were sourced from the reference genome, covering nearly the entire 778 Mb assembly. Only 4.5% were from unmapped scaffolds, de novo transcripts, and BAC end sequences. Consequently, the 690K SNP array was effective for constructing the catfish linkage map, with 287,370 SNPs mapped to 29 linkage groups that align with the haploid chromosome number. This genetic map incorporated 97.8% of the reference genome (1602 scaffolds, 766 Mb) and integrated 1007 previously unmapped scaffolds, facilitating genome-wide recombination analysis [30].

A GWAS study using 900 catfish and a 690K SNP array identified three significant and three suggestive QTLs for resistance to enteric septicemia of catfish (ESC) on LGs 1, 2, 3, 21, and 26. Thirty-seven immune-related genes, including nlrc3 and nlrp12, were identified as candidate ESC resistance genes, confirming one known and identifying five novel QTLs, which support marker-assisted selection [38]. In a study, a team assembled the blue catfish genome, finding that the 690K SNP array had 98.2% alignment with the channel catfish genome but only 23.9% unique hits with the blue catfish genome, yielding 76,399 interspecific informative SNPs sparsely distributed across the genome [36].

## 7. Cross-Application of SNP Arrays for Genomic Analysis

This review discusses 10 publications more accurately on the use of cross-species SNP arrays in mammals, birds, and fish. These studies focused on nine SNP chips, which include five medium-density and four high-density arrays, covering a total of 22 non-target species (Table S2). Additionally, 11 other articles that were less thoroughly reviewed were included to provide a broader perspective through involving seven other SNP chips, encompassing 44 non-target species (excluding the 107 taxa discussed by Miller et al. [28]).

### 7.1. Avian and Mammalian Studies from the 10 Thoroughly Reviewed Publications

The success of cross-species SNP array applications varied significantly based on the phylogenetic relatedness between the target species and the species being genotyped (Table S2). Studies using the BovineSNP50 BeadChip (Illumina, Inc., San Diego, CA, USA) on bison, closely related to cattle, demonstrated high call rates and polymorphism [71]. However, when used on more distantly related species like deer (*Odocoileus* sp.) and reindeer, call rates decreased, though polymorphic SNPs suitable for population genetic analysis were still found in deer [72,73]. In contrast, using caprine and ovine SNP chips on dromedary camels resulted in more limited polymorphic SNPs after quality control [74,75]. The BovineHD BeadChip (Illumina, Inc., San Diego, CA, USA) applied to yak identified significant copy number variation regions (CNVRs) linked to economically important traits and provided moderate results for water buffalo, albeit with lower quality [76,77]. In avian studies, the chicken (*G. g. domesticus*) SNP array gave low call rates and usable SNPs for pigeons (*Columba livia domestica*) but performed better with grouse, yielding significant polymorphic SNPs for phenotypic gene identification [24,78].

### 7.2. Genotyping Fish Species with Medium- and High-Density Arrays

Drywa et al. [79] effectively used a 15K Atlantic salmon array (Illumina, Inc., San Diego, CA, USA) to differentiate populations of sea trout, highlighting the potential for examining closely related species. In contrast, a 250K common carp array (Affymetrix, Inc., Santa Clara, CA, USA) showed polymorphism in related cyprinids; however, substantial filtering due to small sample sizes limited its immediate use [62]. Zhang et al. [80] assessed the Axiom 57K Rainbow Trout Genotyping Array (Affymetrix, Inc., Santa Clara, CA, USA) across seven salmonid species, finding that stringent filtering (e.g., >99% call rate) reduced the dataset to an informative set of 11,643 SNPs for phylogenetic analysis. Similarly, Kim et al. [81] demonstrated the effectiveness of the Axiom 200K Coho Salmon Genotyping Array (Thermo Fisher Scientific, Inc., Santa Clara, CA, USA) for chum salmon (*Oncorhynchus keta*), retaining 1838 SNPs after rigorous filtering (>95% call rate, >5% minor allele frequency) for population structure analysis. These findings emphasize the importance of considering both array design and analytical parameters when employing cross-species genotyping approaches in salmonids. Peñaloza et al. [82] discussed combined-species SNP arrays for aquaculture species, which could have more chance to have more successful usage in cross-species application. Overall, these studies underscore the importance of phylogenetic relatedness and strict filtering strategies in using SNP arrays for non-model organisms.

## 8. Discussion

### 8.1. Advantages and Vulnerabilities of SNP-Based Selection in Catfish

Aquaculture, a rapidly expanding sector of global food production, provided 44% of finfish and 33% of total aquatic animal production in 2022, addressing protein demands and reducing pressure on wild stocks. Projected to grow 10% by 2032, aquaculture is expected to contribute 111 million tons to the total aquatic animal production of 205 million tons [4]. Catfishes, farmed globally for centuries, are a significant component, representing 10.8% of global finfish products in 2022 [4,83]. While breeding programs exist, such as the induced breeding research on European catfish by Szabo et al. [84], many lack the genetic information essential for effective enhancement [3,61]. Despite their importance, catfish breeding programs often lag behind other livestock in utilizing advanced genetic techniques. This gap presents both a challenge and an opportunity to apply modern genomic tools for the sustainable intensification of catfish aquaculture.

#### 8.1.1. Strengths of Genetic-Based Selection

The strengths inherent in the current application of genetic selection are significant. As a research-driven innovation, the market for single nucleotide polymorphisms is divided into two main segments: genotyping (60%) and analysis (40%). Personalized medicine and pharmacogenomics are the key growth drivers in each of these areas. In 2023, the global SNP genotyping and analysis market reached USD 20.2 billion, and it is projected to grow at a 21.9% compound annual growth rate (CAGR), reaching USD 90.7 billion by 2032 [33], fueled by the increasing prevalence of genetic analysis. Diagnostic laboratories represent the largest application sector (50%) for disease-related uses, while research and academic institutions account for another substantial segment (40%) focused on genetic and pharmacological investigations. Additionally, the emerging “Others” category (10%) highlights a growing frontier, particularly with respect to agricultural applications of SNP technology [35]. These advancements significantly contribute to genetic improvements in crops and livestock, showcasing an expanding adoption of these tools beyond traditional healthcare and research fields [33,35].

The established technological framework of SNP genotyping, noted for its cost-effectiveness and efficiency in high-throughput screening, supports its implementation, especially in channel catfish [85]. This technology has consistently yielded substantial genetic gains in crucial traits, including morphology, growth rate, disease resistance (e.g., against *Edwardsiella ictalurid*), and fillet yield [17,38,42,69]. Consequently, the adoption of genetic selection is directly linked to improved productivity and profitability, serving as a primary driver for its ongoing integration. Moreover, ongoing research utilizing WGS and GWAS continues to identify novel genetic markers, thereby refining and expanding the scope of SNP-based selection [32]. A prominent example of a successful breeding program with SNP chip usage in catfish is the interspecific hybridization between channel catfish and blue catfish, which has markedly enhanced catfish production in the United States. F1 hybrids demonstrate superior growth in earthen ponds; however, they experience outbreeding depression when raised in tank environments. Therefore, there remains a need for further improvement of the hybrid due to the occurrence of this environment-dependent heterosis [86]. Similar to this, it is possible to optimize other catfish production across various aquaculture settings through comprehensive genomic and transcriptomic studies.

#### 8.1.2. Weaknesses of SNP-Array Usage in Breeding

The widespread use of genetic selection presents several weaknesses. A significant barrier is the economic feasibility of implementing SNP genotyping, particularly for smaller-scale producers. This is due to the substantial initial investment required for infrastructure and expertise. The costs associated with SNP genotyping platforms—including instruments, reagents, and ongoing expenses for consumables, maintenance, and software—can impose a major financial strain, especially on resource-limited entities. Approximately 20% of academic institutions struggle with acquisition due to these high upfront and recurring costs, often opting for less advanced and more affordable genotyping methods instead [35]. Consequently, the high costs of both the platforms and individual assays can limit access to essential genomic analysis in various research and clinical settings. This restriction hinders broader market growth despite technological advancements aimed at reducing costs and increasing efficiency [33,87]. Moreover, traditional methods are often seen as simpler. Also, the limited availability of genomic resources for non-channel catfish species, such as African catfish (*C. gariepinus*), Bighead catfish (*Clarias macrocephalus*), and European catfish (*S. glanis*) impedes developing and implementing genetic-based species-specific selection strategies. To effectively utilize genetic data, specialized expertise in bioinformatics and statistical genetics is required, which may be lacking in many aquaculture operations. Additionally, there are concerns regarding the potential reduction in genetic diversity due to an over-reliance on elite individuals, which could impact long-term population resilience [22,88]. While acknowledging the benefits of selective breeding, the researchers advise caution against the potential reduction in genetic diversity that can result from intensive selection. Maintaining a broad genetic base is emphasized as crucial for the long-term sustainability of breeding programs, ensuring that populations retain the natural capacity to adapt to diseases or environmental stressors in aquaculture systems [22,89,90].

#### 8.1.3. The Opportunities of Controlled Heredity

Conversely, several opportunities exist to expand the application of genetic selection. The continual reduction in WGS and SNP genotyping costs, driven by technological advancements, enhances accessibility for a broader range of producers. This substantial decline in genome sequencing expenses meant they dropped from approximately USD 1 million in 2007 to around USD 500 by 2023 (in the case of human genome) and potentially USD 200 with advancements like Illumina NovaSeq X (Illumina, Inc., San Diego, CA, USA). This cost reduction renders advanced genomic tools more feasible for agricultural research and development. Consequently, the increased accessibility ensures equitable affordability for agricultural research globally—especially in resource-limited nations—for maximizing the potential of the technology in sustainable food production [91]. Moreover, the low-density SNP panels have been reported to reduce the cost of genotyping [88]. The expansion of genomic resources for non-channel catfish species will facilitate the development of tailored SNP arrays, broadening the applicability of genetic selection.

Recent investigations have significantly expanded the genomic resources available for non-channel catfish species. For instance, a chromosome-level genome assembly for blue catfish (*I. furcatus*) was recently established, providing a foundational resource for species-specific marker. Furthermore, studies have identified extensive single nucleotide polymorphisms within and between blue catfish strains, crucial for the design of tailored SNP arrays development [36]. In another commercially relevant species, the yellow catfish (*Pelteobagrus fulvidraco*), SNPs associated with body length (BL), body weight (BW), and sexual size dimorphism were discovered and validated [32], demonstrating progress in identifying markers for economically important phenotypes in this species. These advancements collectively underscore a growing focus on developing species-specific genomic tools beyond channel catfish, thereby facilitating more targeted and effective genetic selection strategies in diverse aquaculture species. The integration of genetic selection with advanced breeding technologies holds the potential for accelerated genetic improvement [64]. Moreover, the use of SNP arrays can aid in the conservation of wild populations by allowing for the assessment of hybridization rates and the differentiation from escaped farmed fish [92,93]. Moreover, the growing consumer demand for sustainable aquaculture products allows producers to differentiate themselves by adopting genetic selection practices that enhance efficiency and reduce environmental impact (global climate change) [37,90].

#### 8.1.4. Threats of Genetic Breeding Methods

Despite these opportunities, several threats must be addressed. The emergence of novel diseases like the novel herpesvirus (HV) in the specimen of *S. glanis* [94] can undermine the effectiveness of existing disease resistance markers [38], necessitating continuous adaptation of breeding programs [95]. Environmental variability, including climate change and other stressors, can impact the performance of genetically selected catfish. Global warming causes significant temperature changes, particularly in pond farming. In catfish, certain changes can lead to various physiological reactions that affect their growth and survival. An article investigated 33 species of catfish and concluded that these changes influence blood parameters, trigger enzymatic and hormonal responses, and affect oxygen consumption rates. Furthermore, they may influence sound generation and hearing capabilities, alter nutritional needs, and change other phenotypic characteristics [96]. Besides temperature, other environmental stressors also affect catfishes, requiring the development of robust and adaptable breeding strategies [97,98].

Fish welfare and breeding ethics have historically received less emphasis compared to those of terrestrial animals. This disparity can be attributed to varying ethical perspectives and the complexities involved in interpreting pain responses in fish [99]. Nevertheless, there are various welfare issues, including skeletal deformities, cataracts, and heart disease. Moreover, besides the genetic-based selection, the introduction of transgenic fish (e.g., AquAdvantage salmon) and the utilization of triploid fish in aquaculture are rising concerns surrounding early sexual maturation and the detrimental effects of interbreeding between escaped farmed fish and their wild counterparts [100]. In the case of channel catfish, these techniques are already used too, and, because of this, the negative public perceptions of genetically modified or selected aquaculture products could create market barriers for catfish too [39,40]. Ultimately, the issue is that, although consumers are increasingly concerned about the origins of their food and how it is produced, distinguishing between genetic modification and more traditional genetic-based selective breeding can be confusing [41].

### 8.2. Catfish High-Density SNP-Arrays and Their Usage

As previously mentioned, catfish play a crucial role in global food production and environmental management. Given their significance in these domains, the application of advanced technologies is essential for their sustainable development.

Two commercially available high-density SNP arrays (250K and 690K) were developed for *I. punctatus* and *I. furcatus* in 2014 and 2017, representing valuable resources for genetic studies and improvement programs. A medium-density chip was also made beside them when a 55K SNP panel (Thermo Fisher Scientific, Inc., Santa Clara, CA, USA) was employed in channel catfish to pinpoint SNPs associated with harvest and carcass weights [42]. In which study, a specific genomic evaluation using single-step Genomic Best Linear Unbiased Prediction (ssGBLUP) was used, which considerably improved predictive accuracy for these traits, achieving increases of approximately 28% for harvest and 36% for carcass weight compared to traditional methods. The 250K array was developed before the assembly of the reference genome of channel catfish, so it did not cover all parts of the chromosomes. In a GWAS study, this 250K SNP panel was utilized to explore QTL associated with low dissolved oxygen (DO) tolerance in the blue catfish x channel catfish hybrid system [101]. This study identified four linkage clusters, and genes in these regions were involved in different pathways, including mitogen-activated protein kinase (MAPK) and vascular endothelial growth factor (VEGF), shedding light on the complex genetic architecture of hypoxia tolerance in catfish. The 690K array was used on *I. punctatus* and *I. furcatus* genomes to test its ability to detect SNPs in both species. Probe sequences were aligned to channel and blue catfish genomes using an in silico PCR tool, filtering for size and perfect matches. This study successfully proved that a commercial SNP chip is useable for linkage mapping [29,30,36].

These results demonstrate the utility of SNP annotations for improving catfish genomics while supporting preservation and aquaculture breeding programs. However, the Affymetrix Axiom 250K SNP Array and Axiom Catfish 690K Genotyping Array were developed using the available genomic material of *I. punctatus* and *I. furcatus.* Because of this, they are primarily suitable for these species and their F1 hybrids [29,30]. These are the only available high-density SNP arrays, so their cross-species application can be a solution for other catfish species. The 250K chip was validated by wild channel catfish, two blue catfish lines, F1 and F3 backcross hybrids, and two other species (*A. nebulosus* and *A. catus*). The 690K array was assembled to have a broader range of SNPs (e.g., from Thompson, Hatchery, Marion, and USDA 103 commercial lines; Coosa River wild line) to genotype production traits (e.g., growth rates, disease resistance, and adaptability) and to get more diverse markers (6622 SNPs from wild fishes) for population genetics. It is important because even if the domestication of catfishes started ~100 years ago, it could lower genetic diversity by selecting animals with only the desired genotypes. It is likely the reason to when Balog et al. [78] applied a chicken SNP chip containing 57,636 SNPs (Illumina, Inc., San Diego, CA, USA) to squab pigeons—species that diverged around 85 Ma and are domesticated—found 8 SNPs out of 356 that had been previously linked to economically important traits in poultry, such as meat production, feed utilization, and abdominal fat development.

Two other species validated the catfish 250K SNP chip; the brown bullhead and White catfish, which belong to different clades (*Natalis* and *Catus*) in the *Ameiurus* genera under the *Ictaluridae* family [102]. This means they are closer genetically to the channel and Blue catfishes than the *C. gariepinus* (*Clariidae* family) or *S. glanis* (*Siluridae* family). Therefore, these catfish arrays were tested on species from the same genera and their hybrids and closely related species but from different genera.

### 8.3. Cross-Species SNP Array Applications on Different Levels

#### 8.3.1. Species-Level Divergence

We reviewed two literature studies of closely related cross-species SNP application with similar genetic distances: 1. bovine SNP chip on polled yak [77]; 2. coho salmon SNP chip on chum salmon [81] (Table S2).

Due to their close evolutionary relationship [61] (~2 Ma divergence), polled yaks (*B. grunniens*) and cattle share a similar karyotype, making the high-density Illumina BovineHD BeadChip (777,962 SNPs) suitable for generating the first genome-wide CNV map of yaks [77]. This study identified 1066 copy number variation regions spanning 7.2% of the yak autosomal genome, with functional analysis linking associated genes to high-altitude adaptation and economically important traits. Conversely, Kim et al. [81] utilized the Axiom 200K Coho Salmon Genotyping Array on chum salmon which, despite a close relationship (~7 Ma divergence), yielded only 1838 (0.91%) polymorphic SNPs after MAF filtering, likely due to independent evolution or lineage-specific mutations. These contrasting examples highlight the complex relationship between phylogenetic distance, array density, and the success of cross-species SNP array applications. This relationship is evident in the case of the low (7.8%; 3.9%) SNP polymorphism observed when the 250K catfish array was used on blue catfishes, while the wild and backcross hybrids exhibited at least 52.3%. The notably low polymorphism in the *I. furcatus* lines, despite their genetic contribution to the development of the SNP chip, is significant (Table 2 and Table 3). This, along with the relatively significant difference in polymorphism between the Rio Grande and D&B lines, suggests that the genotyped blue catfish likely represents a distant lineage within the species. Besides these studies, vonHoldt et al. [103] used canine arrays on wolves (*Canis lupus*) and dogs (*Canis lupus familiaris*), which are considered subspecies. Thus, while closely related species may facilitate copy number variation detection with high-density arrays, even closely related species can present challenges for SNP discovery with lower-density arrays due to potential species-specific variation.

#### 8.3.2. Genera-Level Divergence

In this comparative SNP array analysis, four instances from the 10 deeply reviewed literature studies exhibited similar genetic distances between target and non-target species to what is between Brown Bullhead, White catfish, and the *Ictalurus* species of the 250K catfish array. These were 1. bovine SNP chip on bison [71]; 2. bovine SNP chip on water buffalo [76]; 3. chicken SNP chip on grouse [24]; and 4. rainbow trout SNP chip on six other Salmonid species [80]. All of these studies are within the same taxonomic family as the SNP was intended for and offer valuable comparative insights. Notably, neither chip had 250K SNP density across these four cases, which makes it challenging to make a statistically acceptable comparison (Table S2).

Pertoldi et al. [71] genotyped bison with a bovine 50K SNP chip (99.57% call rate and 25% MAF). Like the 250K catfish chip, this array was validated with related species: *Bos bison*, *Bos gaurus*, *Bos grunniens*, *Bos javanicus*, *Bubalus depressicornis*, and *Syncerus caffer*. With four species from the same and two from different genera, the bovine chip had 11,206 (20.5%) polymorphic SNPs, with a call rate of 99.3% and 5% MAF. The number of polymorphic SNPs found was similar to that of the brown bullhead catfish (12,649) and white catfish (12,833) in the case of the 250K catfish array. However, their polymorphism rate was significantly higher than that of the catfish (5.1%). In total, 20.5% polymorphic SNPs across related *Bison* species (diverged ~2.7 Ma) were found, though the bison call rate was lower (97.6%) with a different MAF (>0.1%). Borquis et al. [76] used a higher-density (777K) bovine chip on water buffalo (diverged ~7.4 Ma) but obtained far fewer (0.21%) polymorphic SNPs, likely due to greater evolutionary distance, chromosome number differences, and stricter filtering. In 2020, a high-throughput SNP array, NingXin-I with 600K SNPs (Thermo Fisher Scientific, Inc., Santa Clara, CA, USA), was developed for the commercially important large yellow croaker (*Larimichthys crocea*) in China. Evaluation of the array in five closely related *Sciaenidae* species—little yellow croaker (*L. polyactis*), big head croaker (*Collichthys lucidus*), brown croaker (*Miichthys. miiuy*), yellow drum (*Nibea albiflora*), and dusky roncador (*Megalonibea fusca*)—revealed polymorphic SNP rates ranging from 26.68% to 56.23%. The working SNP rate (call rate > 0.9) was high across all six species, ranging from 87.45% in dusky roncador to 95.86% in large yellow croaker. The number of polymorphic SNPs varied considerably among the species, from 154,600 to 483,100, while species-specific SNPs ranged from 3710 to 103,590. This evaluated the SNP array for usage between different species in the family [63]. Xu et al. [62] developed a 250,000 SNP array for common carp (*C. carpio*), and cross-species application to eight related cyprinid species revealed 53,526 to 71,984 polymorphic SNPs per species, suggesting the array’s potential broader utility. However, the authors noted that small sample sizes (5–15 individuals) likely limited the observed polymorphism in related species, emphasizing the need for more extensive studies to assess fully the cross-species applicability of the array. Minias et al. [24] genotyped grouse (diverged ~7.3–37 Ma) with a chicken 580K SNP array (Thermo Fisher Scientific, Inc., Santa Clara, CA, USA), achieving a 69% call rate and identifying species-specific SNPs, though chromosome number variation exists. Zhang et al. [80] used a 57K trout array on various salmonids (diverged 1.5–39 Ma), observing varying call rates and polymorphism, with the highest in close and lowest in more distantly related species, highlighting the impact of divergence. Similar success was seen in other research with ovine array on sheep [104] and bovine array on antelopes [105]. Meanwhile, Drywa et al. [79] selected a diagnostic panel of 39 SNPs (mean FST = 0.1298, MAF > 0.01), which was from the 15K salmon array, and genotyped two Baltic Sea trout populations with it. This study demonstrates the limited, yet still viable, potential of using lower-density, more affordable SNP chips for closely related species.

Therefore, more significant genetic divergence from the reference genome (e.g., channel catfish) used for array design requires a more significant sample number and would result in fewer shared polymorphic SNPs, thus explaining the low polymorphism.

#### 8.3.3. Family-Level Divergence

From the 10 thoroughly studied publications, four studies have explored the use of cross-species SNP arrays among distantly related species from various families. However, these studies reveal significant challenges related to family-level genetic divergence [72,73,78,106], demonstrating that evolutionary distance affects the efficiency and reliability of SNP analysis (Table S2).

For example, Kharzinova et al. [73] applied the BovineSNP50 and OvineSNP50 BeadChips (Illumina, Inc., San Diego, CA, USA) to reindeer (*R. tarandus*). But the *Caprinae* subfamily (sheep and goat) divergence from the *Bovidae* occurred around 19 Ma, resulting in low call rates of 43% and 47%, and limited polymorphism (5.3% and 2%). Similarly, Haynes and Latch [72] used the BovineSNP50 on mule deer, black-tailed deer (*Odocoileus hemionus* ssp.), and white-tailed deer (*Odocoileus virginianus*), which belonged to the *Cervidae* family, yielding a call rate of only 38.7% but identifying 1068 novel SNPs. Bertolini et al. [106] explored the Bovine 777K, Ovine 600K, and Caprine 50K SNP BeadChips (Illumina, Inc., San Diego, CA, USA) (targeted species belong to the *Bovidae*) on dromedary camels (*Camelus dromedarius*), belonging to the *Camelidae* family, which diverged from *Bovidae* around 41.4 Ma. In this case, only the bovine and ovine arrays provided a usable number of polymorphic SNPs (27,585 and 88, respectively). Meanwhile, Balog et al. [78] saw a 47% call rate with a 1% MAF threshold and 356 retained SNPs, some potentially associated with domestication-related traits like meat quality, when applying the Chicken_50K array on domestic pigeons (*Columbidae* family), which diverged from chickens (*Phasianidae* family) over 85 Ma (Table S2).

Haynes and Latch [72] identified 1068 polymorphic SNPs in deer, contrasting with findings by Pertoldi et al. [71], who reported a comparable number in bison but with a higher minor allele frequency (MAF > 0.1%). In a study, More et al. [107], similar to Bertolini et al. [106], successfully used Bovine HD Genotyping Beadchip on another *Camelidae* species (*Lama pacos*) with low thresholds (MAF ≥ 1.25%; conversion rate: 0.008%). These instances illustrate that although cross-species genotyping is viable, it generally yields fewer usable SNPs as phylogenetic distance increases. Miller et al. [28] quantified a 1.5% reduction in successful genotyping for every million years of divergence across 107 taxa utilizing ovine, bovine, and equine SNP chips (Illumina, Inc., San Diego, CA, USA). Moreover, McCue et al. [108] achieved a partial success rate of 25% with an equine array (Illumina, Inc., San Diego, CA, USA) on tapirs and rhinoceroses, which belong to closely related families. Hoffman et al. [109] successfully genotyped seals using a canine array (Illumina, Inc., San Diego, CA, USA) despite the significant evolutionary divergence between the suborders. In 2021, Linsky et al. [110] even used human microarray genotyping technology (Illumina, Inc., San Diego, CA, USA) on the Bornean orangutan (*Pongo pygmaeus*) for SNP discovery. Based on these and the previous studies, it can be concluded that the genetic distance between the target species and the SNP array is as important as making the correct settings for our research. As the above-mentioned research has shown, it is possible to successfully genotype even species from another taxonomy family and with over 30 Ma years of distance. Of course, it is not obviously effective, as seen from the caprine chip, which could not genotype the dromedary camel [106].

If we want to use an existing SNP chip, the first step should be to select which SNP array is the most suitable for our research. The density of the chip depends on what kind of experiment will happen as an HD chip has more applicability (e.g., LD, QTL mapping, and chromosome assembly), but it is costly. These examples underscore the importance of the awareness of available genotyping technologies and updating research methodologies accordingly. Choosing the proper test based on the species is as important as the coverage, as we saw with the chicken and trout SNP arrays. Zhang et al. [80] and Minias et al. [24] could genotype five grouse and six non-target salmonid species with regular settings. Meanwhile, Haynes and Latch [72], Bertolini et al. [106], and Balog et al. [78] must have lowered their filtering thresholds, but there were instances in which they failed to function as intended. One of these settings is the MAF, which is widely used to describe the genetic variability of two-allele SNPs and refers to the frequency of the least common SNP allele. Most of these studies used MAF > 5%, a general value. The MAF mostly lowered under this when the genetic distance was more significant. In summary, the reliability of using species-specific SNP chips on distantly related species is largely affected by genetic divergence. This can lead to ascertainment bias, reduce the levels of polymorphism and call rates, and ultimately skew downstream analyses, resulting in incorrect biological interpretations.

Given the extensive evolutionary divergence (34–83.5 Ma) among the *Ictaluridae*, *Clariidae*, and *Siluridae* catfish families, it is highly possible that reducing the MAF filtering threshold (e.g., to zero) on 250K and 690K catfish genotyping arrays may enhance the success for *C. gariepinus* and *S. glanis*. Based on the literature, they may not have vast potential, a large number of polymorphic SNPs, or a high call rate; however, even with a 1% success rate of polymorphic loci, they will still represent a substantial augmentation of available genetic resources (e.g., new reference genome assembly or low-density SNP panel development), significantly enhancing the scope of genetic analyses possible for those species.

## 9. Conclusions

Catfish species are vital to global aquaculture due to their rapid growth, adaptability, and high-quality meat. However, the lack of genetic information for many species limits effective breeding programs and sustainable practices. While SNP genotyping offers several benefits like improved productivity, it also faces challenges such as high costs and limited genomic resources. However, growth opportunities exist through decreasing costs and developing species-specific arrays. To tackle these challenges, utilizing existing SNP arrays from other catfish species is promising. This review examined examples from the 16 SNP arrays, which included 66 non-target species (not including 107 non-model taxa covered in one of the papers). It emphasizes the potential for applying the Affymetrix Axiom 250K SNP array and the Axiom Catfish 690K Genotyping Array to commercially relevant species such as African and European catfish. Despite an estimated low SNP yield of about 1%, using this array could aid researchers and breeders in improving catfish aquaculture and management.

## Figures and Tables

**Table 1:** SWOT analysis of SNP genotyping in catfish breeding.

Strengths	Weaknesses	Opportunities	Threats
Established technological framework	Cost of implementation	Decreasing costs	Emergence of novel diseases
Demonstrated genetic gains	Limited genomic resources	Development of species-specific SNP arrays	Environmental variability
Increased productivity and profitability	Data analysis and interpretation	Integration with advanced technologies	Regulatory hurdles
Research-driven innovation	Genetic diversity concerns	Increased demand for sustainability	Public perception

**Table 2:** Performance of the 250K catfish SNP chip across species.

	Wild Catfish (n = 192)	Backcross1 (n = 192)	Backcross3 (n = 192)	Blue Catfish1 (n = 10)	Blue Catfish2 (n = 10)	Brown Bullhead Catfish (n = 10)	White Catfish (n = 10)
**SNPs converted**	204,437 (81.7%)	198,583 (79.4%)	218,440 (87.3%)	190,867 (76.3%)	193,039 (77.2%)	126,076 (50.4%)	129,716 (51.9%)
**Polymorphic SNPs**	137,459 (55.0%)	130,685 (52.3%)	156,357 (62.5%)	19,549 (7.8%)	9684 (3.9%)	12,649 (5.1%)	12,833 (5.1%)
**Avg. SNP call rate**	99.4%	99.7%	99.8%	n/a	n/a	n/a	n/a

Backcross1 = F1 generation; Backcross3 = F3 generation; blue catfish1 = Rio Grande; blue catfish2 = D&B); n/a = not available [29].

**Table 3:** Comparison of species-specific SNPs integrated into the arrays based on Liu et al. [29] and Zeng et al. [30].

	250K Catfish Array SNPs		690K Catfish Array SNPs	
**Channel catfish**	182,116	71.6%	581,002	84.10%
**Blue catfish**	31,392	12.6%	44,694	6.47%
**Inter-specific**	39,605	15.8%	64,966	9.40%
**Total SNPs**	250,113	100%	690,662	100%

## Data Availability

No new data were created or analyzed in this study. Data sharing is not applicable to this article.

## Supplementary Materials

The following supporting information can be downloaded at https://www.mdpi.com/article/10.3390/genes16060717/s1. Table S1: Comparison of channel (*Ictalurus punctatus*), blue (*Ictalurus furcatus*), African (*Clarias gariepinu*), and European catfish (*Silurus glanis*) [70,111,112,113,114,115]; Table S2: Comparison of the data on the applicability of SNP chips and the species in the 10 most thoroughly reviewed articles [24,61,71,72,73,76,77,78,80,81,106,116,117,118,119,120,121,122,123,124,125,126,127,128,129,130,131,132,133,134,135,136].

## Abbreviations

The following abbreviations are used in this manuscript:

BL	Body Length
BW	Body Weight
CAGR	Compound Annual Growth Rate
CNV	Copy Number Variation
CNVR	Copy Number Variation Region
DO	Dissolved Oxygen
ESC	Enteric Septicemia of Catfish
FCE	Feed Conversion Efficiency
GMO	Genetically Modified Organism
GS	Genomic Selection
GWAS	Genome-Wide Association Study
HV	Novel Herpesvirus
MAF	Minor Allele Frequency
MAPK	Mitogen-Activated Protein Kinase
MAS	Marker-Assisted Selection
NGS	Next-Generation Sequencing
PCA	Principal Component Analysis
QTL	Quantitative Trait Loci
RAD-Seq	Restriction Site-Associated DNA Sequencing
RNA-Seq	RNA Sequencing
SNP	Single Nucleotide Polymorphism
ssGBLUP	Single-step Genomic Best Linear Unbiased Prediction
USDA-ARS	United States Department of Agriculture—Agricultural Research Service
WARU	Warmwater Aquaculture Research Unit
VEGF	Vascular Endothelial Growth Factor
WGS	Whole-Genome Sequencing
WOS	Web of Science
